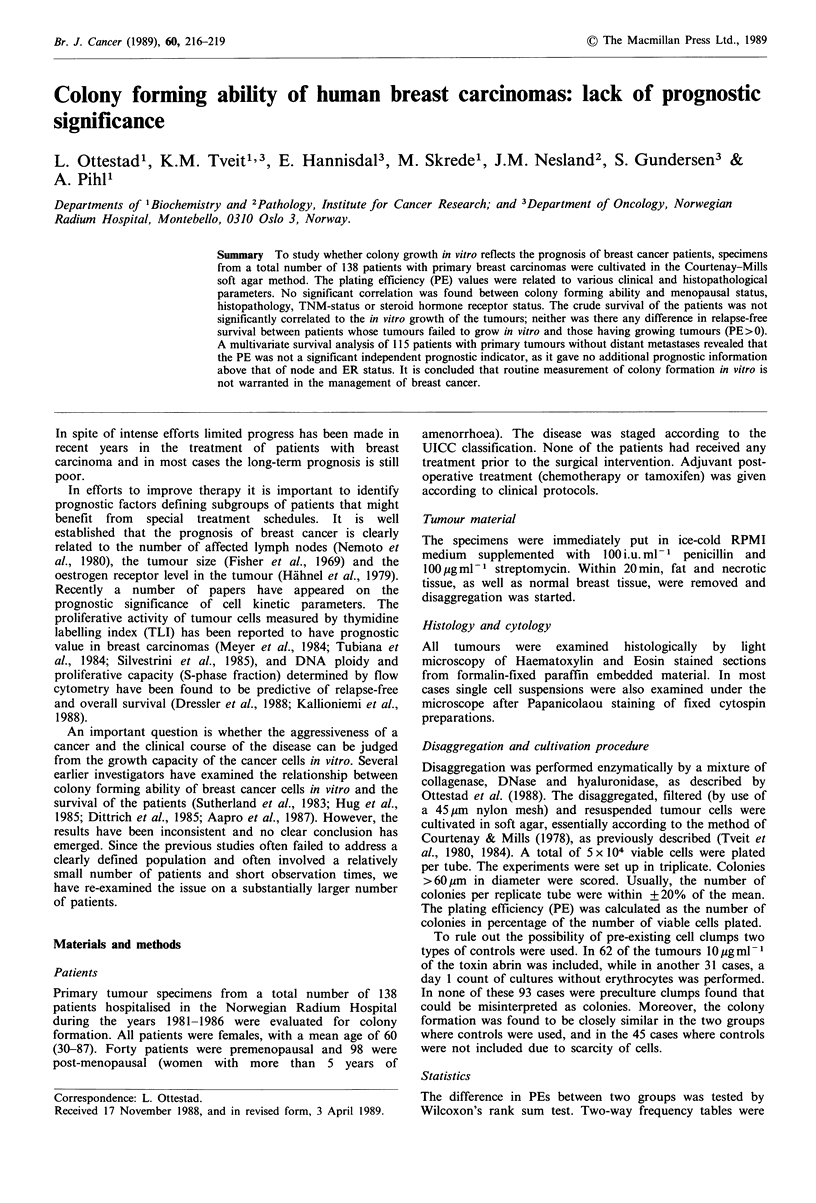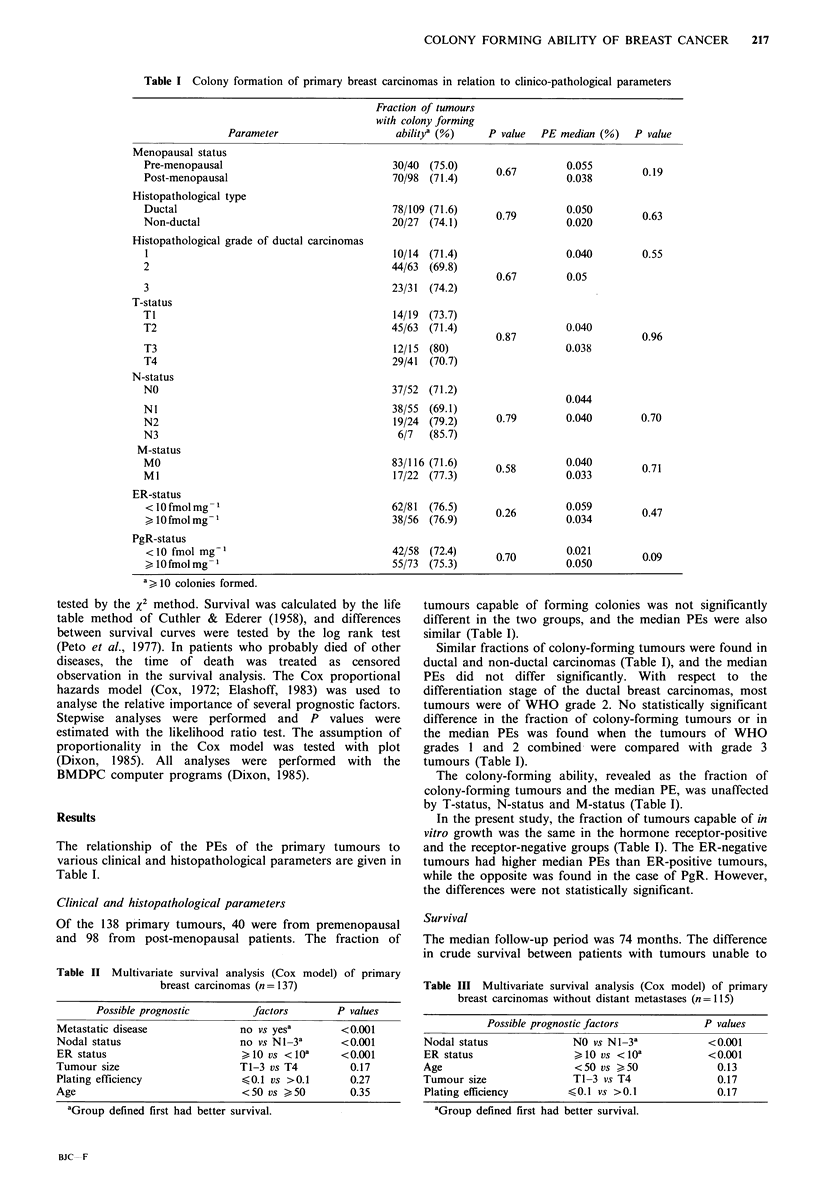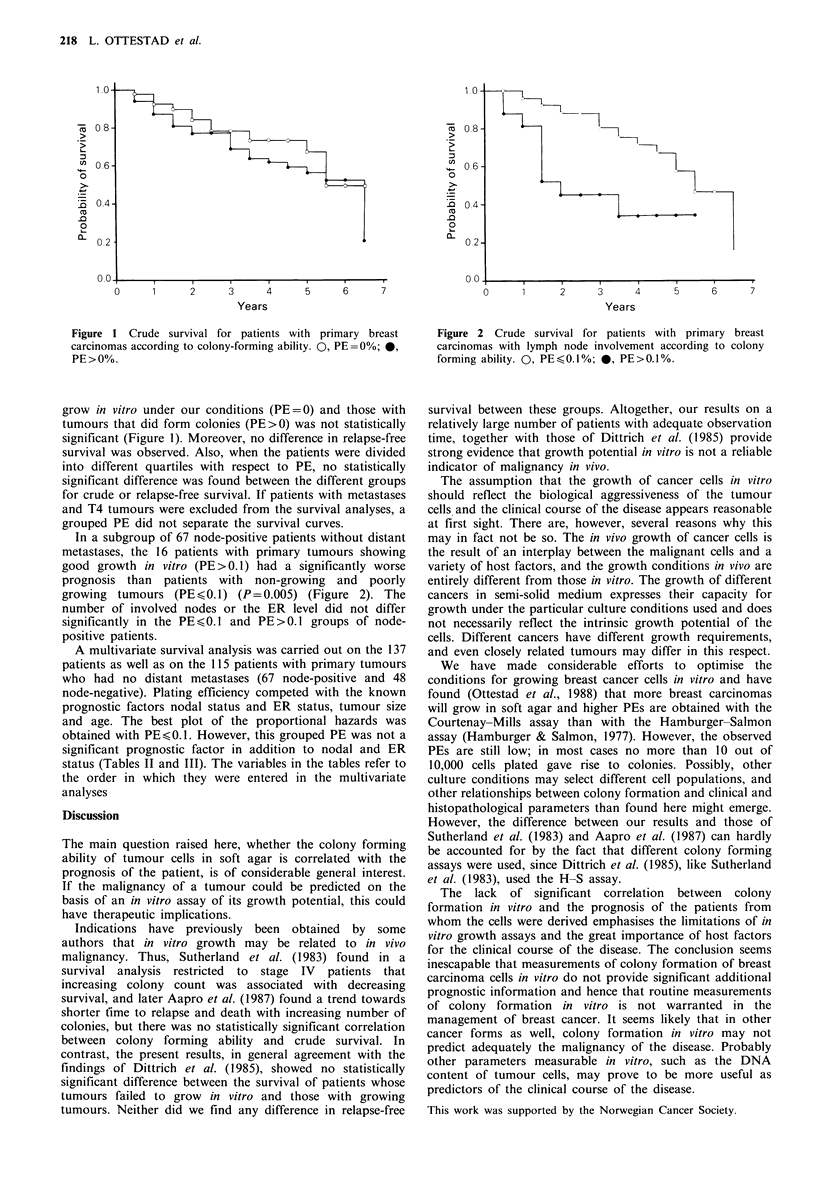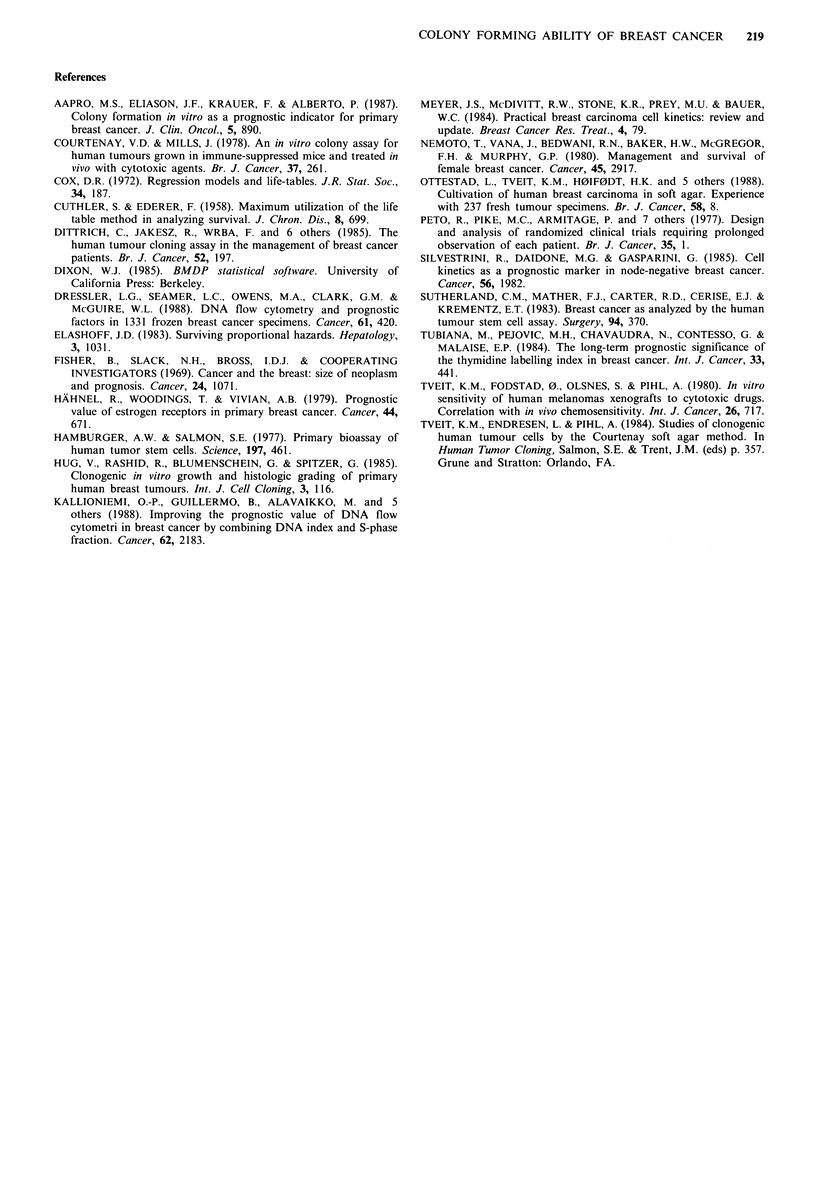# Colony forming ability of human breast carcinomas: lack of prognostic significance.

**DOI:** 10.1038/bjc.1989.254

**Published:** 1989-08

**Authors:** L. Ottestad, K. M. Tveit, E. Hannisdal, M. Skrede, J. M. Nesland, S. Gundersen, A. Pihl

**Affiliations:** Department of Biochemistry, Institute for Cancer Research, Oslo, Norway.

## Abstract

To study whether colony growth in vitro reflects the prognosis of breast cancer patients, specimens from a total number of 138 patients with primary breast carcinomas were cultivated in the Courtenay-Mills soft agar method. The plating efficiency (PE) values were related to various clinical and histopathological parameters. No significant correlation was found between colony forming ability and menopausal status, histopathology, TNM-status or steroid hormone receptor status. The crude survival of the patients was not significantly correlated to the in vitro growth of the tumours; neither was there any difference in relapse-free survival between patients whose tumours failed to grow in vitro and those having growing tumours (PE greater than 0). A multivariate survival analysis of 115 patients with primary tumours without distant metastases revealed that the PE was not a significant independent prognostic indicator, as it gave no additional prognostic information above that of node and ER status. It is concluded that routine measurement of colony formation in vitro is not warranted in the management of breast cancer.


Br. J. Cancer (1989), 60, 216–219 © The Macmillan Press Ltd., 1989

### Colony forming ability of human breast carcinomas: lack of prognostic significance

L. Ottestad[1], K.M. Tveit[1,3], E. Hannisdal[3], M. Skrede[1], J.M. Nesland[2], S. Gundersen[3] & A. Pihl[1]

*Departments of [1]Biochemistry and [2]Pathology, Institute for Cancer Research; and [3]Department of Oncology, Norwegian Radium Hospital, Montebello, 0310 Oslo 3, Norway.*

**Summary** To study whether colony growth *in vitro* reflects the prognosis of breast cancer patients, specimens from a total number of 138 patients with primary breast carcinomas were cultivated in the Courtenay–Mills soft agar method. The plating efficiency (PE) values were related to various clinical and histopathological parameters. No significant correlation was found between colony forming ability and menopausal status, histopathology, TNM-status or steroid hormone receptor status. The crude survival of the patients was not significantly correlated to the *in vitro* growth of the tumours; neither was there any difference in relapse-free survival between patients whose tumours failed to grow *in vitro* and those having growing tumours (PE>0). A multivariate survival analysis of 115 patients with primary tumours without distant metastases revealed that the PE was not a significant independent prognostic indicator, as it gave no additional prognostic information above that of node and ER status. It is concluded that routine measurement of colony formation *in vitro* is not warranted in the management of breast cancer.

In spite of intense efforts limited progress has been made in recent years in the treatment of patients with breast carcinoma and in most cases the long-term prognosis is still poor.

In efforts to improve therapy it is important to identify prognostic factors defining subgroups of patients that might benefit from special treatment schedules. It is well established that the prognosis of breast cancer is clearly related to the number of affected lymph nodes (Nemoto *et al.*, 1980), the tumour size (Fisher *et al.*, 1969) and the oestrogen receptor level in the tumour (Hähnel *et al.*, 1979). Recently a number of papers have appeared on the prognostic significance of cell kinetic parameters. The proliferative activity of tumour cells measured by thymidine labelling index (TLI) has been reported to have prognostic value in breast carcinomas (Meyer *et al.*, 1984; Tubiana *et al.*, 1984; Silvestrini *et al.*, 1985), and DNA ploidy and proliferative capacity (S-phase fraction) determined by flow cytometry have been found to be predictive of relapse-free and overall survival (Dressler *et al.*, 1988; Kallioniemi *et al.*, 1988).

An important question is whether the aggressiveness of a cancer and the clinical course of the disease can be judged from the growth capacity of the cancer cells *in vitro*. Several earlier investigators have examined the relationship between colony forming ability of breast cancer cells *in vitro* and the survival of the patients (Sutherland *et al.*, 1983; Hug *et al.*, 1985; Dittrich *et al.*, 1985; Aapro *et al.*, 1987). However, the results have been inconsistent and no clear conclusion has emerged. Since the previous studies often failed to address a clearly defined population and often involved a relatively small number of patients and short observation times, we have re-examined the issue on a substantially larger number of patients.

### Materials and methods

### *Patients*

Primary tumour specimens from a total number of 138 patients hospitalised in the Norwegian Radium Hospital during the years 1981–1986 were evaluated for colony formation. All patients were females, with a mean age of 60 (30–87). Forty patients were premenopausal and 98 were post-menopausal (women with more than 5 years of amenorrhoea). The disease was staged according to the UICC classification. None of the patients had received any treatment prior to the surgical intervention. Adjuvant post-operative treatment (chemotherapy or tamoxifen) was given according to clinical protocols.

### *Tumour material*

The specimens were immediately put in ice-cold RPMI medium supplemented with 100 i.u. $ml^{-1}$ penicillin and 100 $\mu g\,ml^{-1}$ streptomycin. Within 20 min, fat and necrotic tissue, as well as normal breast tissue, were removed and disaggregation was started.

### *Histology and cytology*

All tumours were examined histologically by light microscopy of Haematoxylin and Eosin stained sections from formalin-fixed paraffin embedded material. In most cases single cell suspensions were also examined under the microscope after Papanicolaou staining of fixed cytospin preparations.

### *Disaggregation and cultivation procedure*

Disaggregation was performed enzymatically by a mixture of collagenase, DNase and hyaluronidase, as described by Ottestad *et al.* (1988). The disaggregated, filtered (by use of a 45 $\mu$m nylon mesh) and resuspended tumour cells were cultivated in soft agar, essentially according to the method of Courtenay & Mills (1978), as previously described (Tveit *et al.*, 1980, 1984). A total of $5\times10^4$ viable cells were plated per tube. The experiments were set up in triplicate. Colonies $>60\,\mu$m in diameter were scored. Usually, the number of colonies per replicate tube were within $\pm20\%$ of the mean. The plating efficiency (PE) was calculated as the number of colonies in percentage of the number of viable cells plated.

To rule out the possibility of pre-existing cell clumps two types of controls were used. In 62 of the tumours 10 $\mu g\,ml^{-1}$ of the toxin abrin was included, while in another 31 cases, a day 1 count of cultures without erythrocytes was performed. In none of these 93 cases were preculture clumps found that could be misinterpreted as colonies. Moreover, the colony formation was found to be closely similar in the two groups where controls were used, and in the 45 cases where controls were not included due to scarcity of cells.

### *Statistics*

The difference in PEs between two groups was tested by Wilcoxon's rank sum test. Two-way frequency tables were

Correspondence: L. Ottestad.
Received 17 November 1988, and in revised form, 3 April 1989.

**Table I** Colony formation of primary breast carcinomas in relation to clinico-pathological parameters

| *Parameter* | *Fraction of tumours with colony forming ability*[a] *(%)* | *P value* | *PE median (%)* | *P value* |
|---|---|---|---|---|
| Menopausal status | | | | |
| Pre-menopausal | 30/40 (75.0) | 0.67 | 0.055 | 0.19 |
| Post-menopausal | 70/98 (71.4) | | 0.038 | |
| Histopathological type | | | | |
| Ductal | 78/109 (71.6) | 0.79 | 0.050 | 0.63 |
| Non-ductal | 20/27 (74.1) | | 0.020 | |
| Histopathological grade of ductal carcinomas | | | | |
| 1 | 10/14 (71.4) | | 0.040 | 0.55 |
| 2 | 44/63 (69.8) | 0.67 | 0.05 | |
| 3 | 23/31 (74.2) | | | |
| T-status | | | | |
| T1 | 14/19 (73.7) | | | |
| T2 | 45/63 (71.4) | 0.87 | 0.040 | 0.96 |
| T3 | 12/15 (80) | | 0.038 | |
| T4 | 29/41 (70.7) | | | |
| N-status | | | | |
| N0 | 37/52 (71.2) | | 0.044 | |
| N1 | 38/55 (69.1) | | | |
| N2 | 19/24 (79.2) | 0.79 | 0.040 | 0.70 |
| N3 | 6/7 (85.7) | | | |
| M-status | | | | |
| M0 | 83/116 (71.6) | 0.58 | 0.040 | 0.71 |
| M1 | 17/22 (77.3) | | 0.033 | |
| ER-status | | | | |
| $<10\,\mathrm{fmol\,mg^{-1}}$ | 62/81 (76.5) | 0.26 | 0.059 | 0.47 |
| $\geqslant 10\,\mathrm{fmol\,mg^{-1}}$ | 38/56 (76.9) | | 0.034 | |
| PgR-status | | | | |
| $<10\,\mathrm{fmol\,mg^{-1}}$ | 42/58 (72.4) | 0.70 | 0.021 | 0.09 |
| $\geqslant 10\,\mathrm{fmol\,mg^{-1}}$ | 55/73 (75.3) | | 0.050 | |

[a]$\geqslant$10 colonies formed.

tested by the $\chi^2$ method. Survival was calculated by the life table method of Cuthler & Ederer (1958), and differences between survival curves were tested by the log rank test (Peto *et al.*, 1977). In patients who probably died of other diseases, the time of death was treated as censored observation in the survival analysis. The Cox proportional hazards model (Cox, 1972; Elashoff, 1983) was used to analyse the relative importance of several prognostic factors. Stepwise analyses were performed and *P* values were estimated with the likelihood ratio test. The assumption of proportionality in the Cox model was tested with plot (Dixon, 1985). All analyses were performed with the BMDPC computer programs (Dixon, 1985).

### Results

The relationship of the PEs of the primary tumours to various clinical and histopathological parameters are given in Table I.

### *Clinical and histopathological parameters*

Of the 138 primary tumours, 40 were from premenopausal and 98 from post-menopausal patients. The fraction of tumours capable of forming colonies was not significantly different in the two groups, and the median PEs were also similar (Table I).

Similar fractions of colony-forming tumours were found in ductal and non-ductal carcinomas (Table I), and the median PEs did not differ significantly. With respect to the differentiation stage of the ductal breast carcinomas, most tumours were of WHO grade 2. No statistically significant difference in the fraction of colony-forming tumours or in the median PEs was found when the tumours of WHO grades 1 and 2 combined were compared with grade 3 tumours (Table I).

The colony-forming ability, revealed as the fraction of colony-forming tumours and the median PE, was unaffected by T-status, N-status and M-status (Table I).

In the present study, the fraction of tumours capable of *in vitro* growth was the same in the hormone receptor-positive and the receptor-negative groups (Table I). The ER-negative tumours had higher median PEs than ER-positive tumours, while the opposite was found in the case of PgR. However, the differences were not statistically significant.

### *Survival*

The median follow-up period was 74 months. The difference in crude survival between patients with tumours unable to

**Table II** Multivariate survival analysis (Cox model) of primary breast carcinomas ($n=137$)

| *Possible prognostic* | *factors* | *P values* |
|---|---|---|
| Metastatic disease | no *vs* yes[a] | <0.001 |
| Nodal status | no *vs* N1–3[a] | <0.001 |
| ER status | $\geqslant$10 *vs* <10[a] | <0.001 |
| Tumour size | T1–3 *vs* T4 | 0.17 |
| Plating efficiency | $\leqslant$0.1 *vs* >0.1 | 0.27 |
| Age | <50 *vs* $\geqslant$50 | 0.35 |

[a]Group defined first had better survival.

**Table III** Multivariate survival analysis (Cox model) of primary breast carcinomas without distant metastases ($n=115$)

| *Possible* | *prognostic factors* | *P values* |
|---|---|---|
| Nodal status | N0 *vs* N1–3[a] | <0.001 |
| ER status | $\geqslant$10 *vs* <10[a] | <0.001 |
| Age | <50 *vs* $\geqslant$50 | 0.13 |
| Tumour size | T1–3 *vs* T4 | 0.17 |
| Plating efficiency | $\leqslant$0.1 *vs* >0.1 | 0.17 |

[a]Group defined first had better survival.

**Figure 1** Crude survival for patients with primary breast carcinomas according to colony-forming ability. ○, PE=0%; ●, PE>0%.

**Figure 2** Crude survival for patients with primary breast carcinomas with lymph node involvement according to colony forming ability. ○, PE⩽0.1%; ●, PE>0.1%.

grow *in vitro* under our conditions (PE=0) and those with tumours that did form colonies (PE>0) was not statistically significant (Figure 1). Moreover, no difference in relapse-free survival was observed. Also, when the patients were divided into different quartiles with respect to PE, no statistically significant difference was found between the different groups for crude or relapse-free survival. If patients with metastases and T4 tumours were excluded from the survival analyses, a grouped PE did not separate the survival curves.

In a subgroup of 67 node-positive patients without distant metastases, the 16 patients with primary tumours showing good growth *in vitro* (PE>0.1) had a significantly worse prognosis than patients with non-growing and poorly growing tumours (PE⩽0.1) ($P$=0.005) (Figure 2). The number of involved nodes or the ER level did not differ significantly in the PE⩽0.1 and PE>0.1 groups of node-positive patients.

A multivariate survival analysis was carried out on the 137 patients as well as on the 115 patients with primary tumours who had no distant metastases (67 node-positive and 48 node-negative). Plating efficiency competed with the known prognostic factors nodal status and ER status, tumour size and age. The best plot of the proportional hazards was obtained with PE⩽0.1. However, this grouped PE was not a significant prognostic factor in addition to nodal and ER status (Tables II and III). The variables in the tables refer to the order in which they were entered in the multivariate analyses

### Discussion

The main question raised here, whether the colony forming ability of tumour cells in soft agar is correlated with the prognosis of the patient, is of considerable general interest. If the malignancy of a tumour could be predicted on the basis of an *in vitro* assay of its growth potential, this could have therapeutic implications.

Indications have previously been obtained by some authors that *in vitro* growth may be related to *in vivo* malignancy. Thus, Sutherland *et al.* (1983) found in a survival analysis restricted to stage IV patients that increasing colony count was associated with decreasing survival, and later Aapro *et al.* (1987) found a trend towards shorter time to relapse and death with increasing number of colonies, but there was no statistically significant correlation between colony forming ability and crude survival. In contrast, the present results, in general agreement with the findings of Dittrich *et al.* (1985), showed no statistically significant difference between the survival of patients whose tumours failed to grow *in vitro* and those with growing tumours. Neither did we find any difference in relapse-free survival between these groups. Altogether, our results on a relatively large number of patients with adequate observation time, together with those of Dittrich *et al.* (1985) provide strong evidence that growth potential *in vitro* is not a reliable indicator of malignancy *in vivo*.

The assumption that the growth of cancer cells *in vitro* should reflect the biological aggressiveness of the tumour cells and the clinical course of the disease appears reasonable at first sight. There are, however, several reasons why this may in fact not be so. The *in vivo* growth of cancer cells is the result of an interplay between the malignant cells and a variety of host factors, and the growth conditions *in vivo* are entirely different from those *in vitro*. The growth of different cancers in semi-solid medium expresses their capacity for growth under the particular culture conditions used and does not necessarily reflect the intrinsic growth potential of the cells. Different cancers have different growth requirements, and even closely related tumours may differ in this respect.

We have made considerable efforts to optimise the conditions for growing breast cancer cells *in vitro* and have found (Ottestad *et al.*, 1988) that more breast carcinomas will grow in soft agar and higher PEs are obtained with the Courtenay–Mills assay than with the Hamburger–Salmon assay (Hamburger & Salmon, 1977). However, the observed PEs are still low; in most cases no more than 10 out of 10,000 cells plated gave rise to colonies. Possibly, other culture conditions may select different cell populations, and other relationships between colony formation and clinical and histopathological parameters than found here might emerge. However, the difference between our results and those of Sutherland *et al.* (1983) and Aapro *et al.* (1987) can hardly be accounted for by the fact that different colony forming assays were used, since Dittrich *et al.* (1985), like Sutherland *et al.* (1983), used the H–S assay.

The lack of significant correlation between colony formation *in vitro* and the prognosis of the patients from whom the cells were derived emphasises the limitations of *in vitro* growth assays and the great importance of host factors for the clinical course of the disease. The conclusion seems inescapable that measurements of colony formation of breast carcinoma cells *in vitro* do not provide significant additional prognostic information and hence that routine measurements of colony formation *in vitro* is not warranted in the management of breast cancer. It seems likely that in other cancer forms as well, colony formation *in vitro* may not predict adequately the malignancy of the disease. Probably other parameters measurable *in vitro*, such as the DNA content of tumour cells, may prove to be more useful as predictors of the clinical course of the disease.

This work was supported by the Norwegian Cancer Society.

### References

AAPRO, M.S., ELIASON, J.F., KRAUER, F. & ALBERTO, P. (1987). Colony formation *in vitro* as a prognostic indicator for primary breast cancer. *J. Clin. Oncol.*, **5**, 890.

COURTENAY, V.D. & MILLS, J. (1978). An *in vitro* colony assay for human tumours grown in immune-suppressed mice and treated *in vivo* with cytotoxic agents. *Br. J. Cancer*, **37**, 261.

COX, D.R. (1972). Regression models and life-tables. *J.R. Stat. Soc.*, **34**, 187.

CUTHLER, S. & EDERER, F. (1958). Maximum utilization of the life table method in analyzing survival. *J. Chron. Dis.*, **8**, 699.

DITTRICH, C., JAKESZ, R., WRBA, F. and 6 others (1985). The human tumour cloning assay in the management of breast cancer patients. *Br. J. Cancer*, **52**, 197.

DIXON, W.J. (1985). *BMDP statistical software*. University of California Press: Berkeley.

DRESSLER, L.G., SEAMER, L.C., OWENS, M.A., CLARK, G.M. & McGUIRE, W.L. (1988). DNA flow cytometry and prognostic factors in 1331 frozen breast cancer specimens. *Cancer*, **61**, 420.

ELASHOFF, J.D. (1983). Surviving proportional hazards. *Hepatology*, **3**, 1031.

FISHER, B., SLACK, N.H., BROSS, I.D.J. & COOPERATING INVESTIGATORS (1969). Cancer and the breast: size of neoplasm and prognosis. *Cancer*, **24**, 1071.

HÄHNEL, R., WOODINGS, T. & VIVIAN, A.B. (1979). Prognostic value of estrogen receptors in primary breast cancer. *Cancer*, **44**, 671.

HAMBURGER, A.W. & SALMON, S.E. (1977). Primary bioassay of human tumor stem cells. *Science*, **197**, 461.

HUG, V., RASHID, R., BLUMENSCHEIN, G. & SPITZER, G. (1985). Clonogenic *in vitro* growth and histologic grading of primary human breast tumours. *Int. J. Cell Cloning*, **3**, 116.

KALLIONIEMI, O.-P., GUILLERMO, B., ALAVAIKKO, M. and 5 others (1988). Improving the prognostic value of DNA flow cytometri in breast cancer by combining DNA index and S-phase fraction. *Cancer*, **62**, 2183.

MEYER, J.S., McDIVITT, R.W., STONE, K.R., PREY, M.U. & BAUER, W.C. (1984). Practical breast carcinoma cell kinetics: review and update. *Breast Cancer Res. Treat.*, **4**, 79.

NEMOTO, T., VANA, J., BEDWANI, R.N., BAKER, H.W., McGREGOR, F.H. & MURPHY, G.P. (1980). Management and survival of female breast cancer. *Cancer*, **45**, 2917.

OTTESTAD, L., TVEIT, K.M., HØIFØDT, H.K. and 5 others (1988). Cultivation of human breast carcinoma in soft agar. Experience with 237 fresh tumour specimens. *Br. J. Cancer*, **58**, 8.

PETO, R., PIKE, M.C., ARMITAGE, P. and 7 others (1977). Design and analysis of randomized clinical trials requiring prolonged observation of each patient. *Br. J. Cancer*, **35**, 1.

SILVESTRINI, R., DAIDONE, M.G. & GASPARINI, G. (1985). Cell kinetics as a prognostic marker in node-negative breast cancer. *Cancer*, **56**, 1982.

SUTHERLAND, C.M., MATHER, F.J., CARTER, R.D., CERISE, E.J. & KREMENTZ, E.T. (1983). Breast cancer as analyzed by the human tumour stem cell assay. *Surgery*, **94**, 370.

TUBIANA, M., PEJOVIC, M.H., CHAVAUDRA, N., CONTESSO, G. & MALAISE, E.P. (1984). The long-term prognostic significance of the thymidine labelling index in breast cancer. *Int. J. Cancer*, **33**, 441.

TVEIT, K.M., FODSTAD, Ø., OLSNES, S. & PIHL, A. (1980). *In vitro* sensitivity of human melanomas xenografts to cytotoxic drugs. Correlation with *in vivo* chemosensitivity. *Int. J. Cancer*, **26**, 717.

TVEIT, K.M., ENDRESEN, L. & PIHL, A. (1984). Studies of clonogenic human tumour cells by the Courtenay soft agar method. In *Human Tumor Cloning,* Salmon, S.E. & Trent, J.M. (eds) p. 357. Grune and Stratton: Orlando, FA.